# Disentangling the strings that organize behavior

**DOI:** 10.7554/eLife.38410

**Published:** 2018-06-26

**Authors:** Matthieu Louis, Julie H Simpson

**Affiliations:** 1 Department of Molecular, Cellular and Development BiologyUniversity of California Santa BarbaraSanta BarbaraUnited States; 2 Department of PhysicsUniversity of California, Santa BarbaraSanta BarbaraUnited States; 3 Neuroscience Research InstituteUniversity of California, Santa BarbaraSanta BarbaraUnited States

**Keywords:** descending neurons, sensory-motor, anatomy, split-GAL4, ventral nerve cord, brain, *D. melanogaster*

## Abstract

The neurons that connect the brain and ventral nerve cord in fruit flies have been mapped in unprecedented detail.

**Related research article** Namiki S, Dickinson MH, Wong AM, Korff W, Card GM. 2018. The functional organization of descending sensory-motor pathways in *Drosophila*. *eLife* **7**:e34272. doi: 10.7554/eLife.34272

**Related research article** Cande J, Namiki S, Qiu J, Korff W, Card GM, Shaevitz JW, Stern DL, Berman GJ. 2018. Optogenetic dissection of descending behavioral control in *Drosophila*. *eLife* **7**:e34275. doi: 10.7554/eLife.34275

The nervous systems of animals are typically divided into two parts: a brain in the head and either a ventral nerve cord or a spinal cord in the body. Each plays a role in helping the animal to navigate and interact with its environment. The brain combines information from a number of sources – including the senses, contextual information and the internal state of the animal – to select an appropriate behavior. The ventral nerve cord then coordinates the activity of motor neurons to perform the particular action selected by the brain.

Descending neurons connect the brain to the ventral nerve cord, and it has been estimated that at most 550 pairs of descending neurons exist in adult *Drosophila* fruit flies (Hsu and Bhandawat, 2016). Reaching a better understanding of the anatomy of these descending neurons is a necessary step toward describing the logic of behavioral control. Now, in eLife, Gwyneth Card of the Janelia Research Campus and colleagues – including Shigehiro Namiki as first author, Michael Dickinson, Allan Wong and Wyatt Korff – report a major advance toward this goal by working out a detailed map that traces the path of nearly half of the descending neurons (Namiki et al., 2018). In a second paper David Stern of Janelia, Gordon Berman of Emory University and colleagues – including Jessica Cande as first author, Namiki, Jirui Qui, Korff, Card and Joshua Shaevitz – report the results of experiments on behaviors associated with these descending neurons (Cande et al., 2018).

Using cutting-edge genetic tools, Namiki et al. labeled 190 pairs of descending neurons covering 54 distinct cell types. This allowed the neurons to be mapped from their origin in the brain to their destination in the ventral nerve cord. This anatomical analysis is rare in its depth and coverage. Although the function of a neural circuit cannot be unraveled from its wiring diagram alone (Bargmann and Marder, 2013), such knowledge does help to formulate testable hypotheses about the function of the circuit (Takemura et al., 2013; Takemura et al., 2017).

One striking finding reported by Namiki et al. is that descending neurons tend to be individual and unique rather than members of large populations of neurons with similar structures. An attempt to classify these neurons based on the location of their cell bodies, or where in the brain their inputs come from, was not particularly conclusive: neurons with distinct shapes and neurotransmitter types can have adjacent cell bodies. However, grouping the descending neurons based on the areas of the ventral nerve cord they target led to more coherent results. One group of descending neurons innervates the area of the ventral nerve cord that controls the legs, while another targets the nerves that connect to the wing, and a smaller group connects to an intermediate region called the tectulum (Court et al., 2017). This organization of the descending neurons supports a model in which *Drosophila* has separate streams of control for behaviors associated with its legs (such as walking) and its wings (such as flying or producing courtship songs).

On average, descending neurons connect three regions of the brain to two regions of the ventral nerve cord. The fact that most descending neurons receive and make so few connections suggests that sensory information is processed before it reaches them. Furthermore, it suggests that complex motor outputs are put into action downstream from the descending neurons. These hypotheses are corroborated by the results reported by Cande et al., who used optogenetics and sophisticated behavioral analysis to explore the behaviors elicited by different descending neurons. Activating some individual descending neurons produced simple and repetitive behaviors, but the same general class of behavior (such as walking or grooming) could be evoked by multiple different descending neurons.

Surprisingly, Namiki et al. report that descending neurons do not project exclusively into the ventral nerve cord: the majority of them take a detour through a region of the brain called the subesophageal zone. This zone, which is located under the jaw, processes taste and contains the motor neurons for extending the proboscis (Ito et al., 2014; Kendroud et al., 2018). Circuits in the subesophageal zone of larvae appear to integrate inputs from multiple senses to control how the larvae stop and start as they navigate toward a goal (Tastekin et al., 2015). The anatomical observations of Namiki et al. suggest that this area of the fly brain deserves further study.

Do neurons convey ‘decisions’ from the brain that activate pre-determined motor programs in the ventral nerve cord? According to the debated command neuron model, specific descending neurons would trigger whole coherent behavioral programs, such as courtship or grooming (Kristan, 2008). Although some behaviors might be controlled in this way (Bidaye et al., 2014; von Reyn et al., 2014), such cases are rare and cannot explain the range of complex actions a fly exhibits. Premotor circuits forming central pattern generators in the ventral nerve cord are sufficient to enable decapitated flies and ‘brainless’ larvae to execute coordinated motor programs (Berni et al., 2012; Harris et al., 2015). It is likely that a fly combines the activity of different descending neurons to select and combine the behaviors executed by central pattern generators.

How then is the full repertoire of behaviors directed through the descending neurons? A skilled puppeteer can give ‘life’ to a marionette by pulling on a few strings connected to individual limb joints. The nervous system of an animal faces an analogous challenge: sensory information integrated in the brain has to be conveyed to the motor neurons and muscles through a limited number of descending neurons. Namiki et al. reveals a comprehensive look at these ‘strings’ in *Drosophila*, and Cande et al. demonstrates what can happen when you pull on them, giving us the first steps toward disentangling the logic of behavior control (Figure 1).

**Figure 1.**
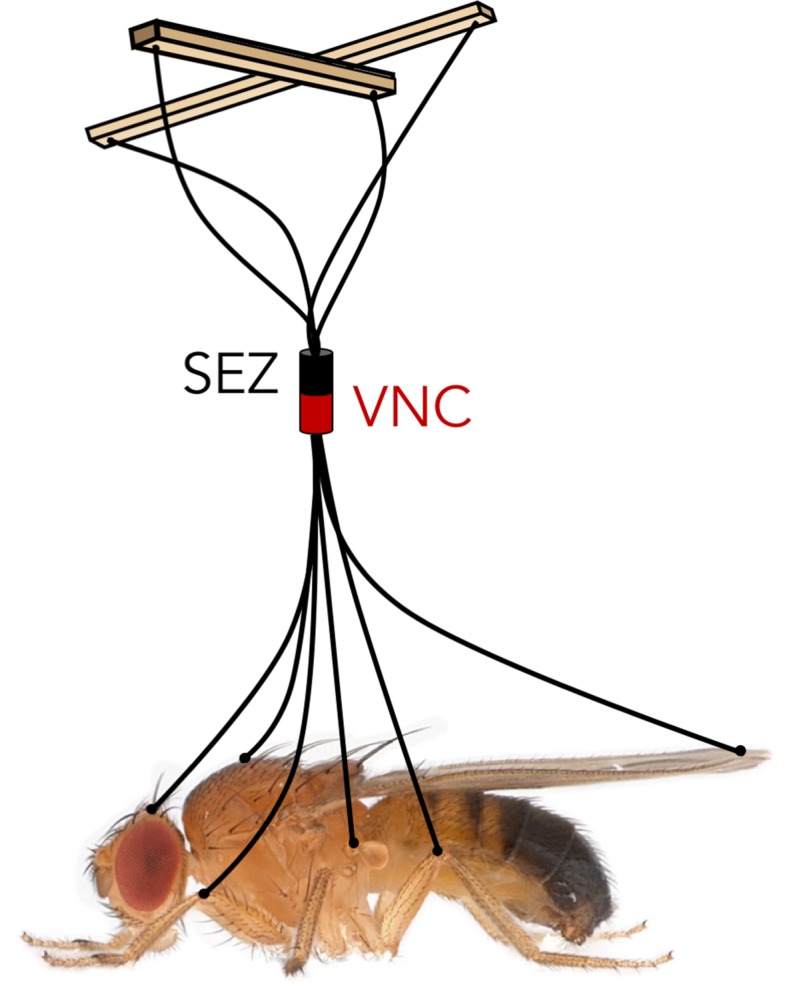
Like controlling the movements of a puppet using only a few strings, the fly brain must control the entire behavioral repertoire of the fly based on the information transmitted by a small set of descending neurons. SEZ: subesophageal zone. VNC: ventral nerve cord.

IMAGE CREDIT: Matthieu Louis and Julie H Simpson, using a photograph of *Drosophila melanogaster* from Nicolas Gompel.
